# Cross-sectional analysis of baseline differences of candidates for rotator cuff surgery: a sex and gender perspective

**DOI:** 10.1186/1471-2474-10-26

**Published:** 2009-02-24

**Authors:** Helen Razmjou, Aileen M Davis, Susan B Jaglal, Richard Holtby, Robin R Richards

**Affiliations:** 1 Holland Orthopaedic & Arthritic Centre, Sunnybrook Health Sciences Centre, Toronto, Canada; 2 Department of Surgery, Sunnybrook Health Sciences Centre, Toronto, Canada; 3 Department of Surgery, University of Toronto, Toronto, Canada; 4 Department of Physical Therapy, University of Toronto, Toronto, Canada; 5 Graduate Departments of Rehabilitation Science and Health Policy, Management and Evaluation, University of Toronto, Toronto, Canada; 6 Division of Health Care and Outcomes Research and the Arthritis Community Evaluation Unit, Toronto Western Research Institute, Toronto, Canada

## Abstract

**Background:**

The word "sex" refers to biological differences between men and women. Gender refers to roles, behaviors, activities, and attributes that a given society considers appropriate for men and women. Traditionally, treatment decisions have been based on patient's sex without including the gender. Assessment of disability secondary to musculoskeletal problems would not be complete or accurate unless potentially relevant biological and non-biological aspects of being a man or woman are taken into consideration. The purposes of this study were to: 1) investigate the difference in pre-operative characteristics between men and women who were candidates for rotator cuff surgery; and, 2) assess the relationship between level of disability and factors that represent sex and factors that signify gender.

**Method:**

This was a cross-sectional study. The primary outcome measure of disability was a disease-specific outcome measure, the Western Ontario Rotator Cuff (WORC) index, and independent variables were sex, age, hand dominance, shoulder side involvement, BMI, co-morbidity, medication use, work status, smoking habits, strength, range of motion, level of pathology, concurrent osteoarthritis, expectations for recovery, and participation restriction. Parametric, non-parametric, univariable, subgroup, and multivariable analyses were conducted.

**Results:**

One hundred and seventy patients were included in the study. The mean age was 57 ± 11, 85 were females. Women reported higher levels of disability despite similar or lower levels of pathology. Scores of the WORC were strongly influenced by factors that represented "gender" such as participation restriction (F = 28.91, p < 0.0001) and expectations for improved activities of daily living (F = 5.80, p = 0.004). Painfree combined range of motion, which represented an interaction between "sex" and "gender" was also associated with disability after being adjusted for all other relevant baseline factors (F = 25.82, p < 0.0001).

**Conclusion:**

Gender-related factors such as expectations and participation limitations have an independent impact on disability in men and women undergoing rotator cuff related surgery.

## Background

The word "sex" refers to those differences that can be attributed to biological differences (e.g., body size and shape, hormonal activity or functioning of organs) between men and women [1]. The word "gender" refers to non-biological characteristics of maleness/femaleness and describes "socially constructed roles and relationships, personality traits, attitudes, behaviors, values, relative power and influence that society ascribes to two sexes based on a differential basis" [2]. While sex is a universal condition of humans, gender roles vary across cultures [3,4]. Traditionally, diagnostic and treatment decisions have been based on patient's sex without including the gender differences that are shaped and influenced by family and society [5-7]. Assessment of disability secondary to musculoskeletal problems would not be complete or accurate unless potentially relevant biological and non-biological aspects of being a man or woman are taken into consideration.

Sex and gender are conceptually related. However, they are two distinct constructs and interchangeable use of these terms has the potential to affect research quality and clarity. Sex/gender-sensitive health research may help clinicians and researchers understand the complexity and diversity of human health by linking biological, psychological, social and cultural factors. Sex/gender sensitive research involves investigating how sex and gender interact with one another to create potentially serious health conditions for which there are distinct risk factors for women or men [2].

The prevalence of musculoskeletal disorders appears to be higher in women [8-12]. In the area of rotator cuff disease, sex or gender related studies have seldom been conducted [13-15] and those that do tend to simply evaluate the effect of males as compared to females. Razmjou et al [13] in a cross-sectional study of surgical candidates for rotator cuff surgery found that women with rotator cuff pathologies had more frustration, depression and worry because of their shoulder problems. Bassey and colleagues [15] reported that women with rotator cuff related pathologies had significantly reduced shoulder abduction. Romeo et al. [14] found that disability as defined by the subjective shoulder measures of Constant-Murley and Simple Shoulder Test (SST) was negatively correlated with age in women but not in men. The results of the limited previous studies do not provide suggestions on the relationship between disability and factors that define sex or gender. The retrospective nature of the studies, unequal sample sizes, and the fact that differentiating between sex and gender related factors was not the primary objective contribute to inconclusive results of these studies. In addition, we are not aware of any gender-sensitive analysis that has examined the complex interactive and combined role of sex and gender on disability. Further study of this subject is therefore warranted as such research may have valuable implications for clinicians, researchers, and policy makers in terms of providing optimal care to both female and male patients suffering from common musculoskeletal disorders. By identifying non-biological factors that affect men/women's disability on differential basis, the development of more cost effective and focused treatment plans will be encouraged.

We hypothesized that women would report higher levels of disability as defined by subjective outcome measures and that gender-related factors would have an independent relationship with disability. Therefore, the purposes of this study were to: 1) investigate the difference in pre-operative characteristics between men and women candidates for rotator cuff related surgery and 2) assess the relationship between level of disability and factors that represented sex and gender.

## Methods

The present study was a cross-sectional analysis of baseline data of a prospective study of patients undergoing rotator cuff related surgery. The target sample was patients referred to one of two surgeons with subspecialty interest in shoulder and upper extremity reconstruction surgery in a large academic institution. Surgical candidates who met the eligibility criteria were approached to participate in the study. In addition to informed consent, the inclusion criteria included age ≥ 18 years, a diagnosis of impingement syndrome and/or rotator cuff disease, and unremitting pain in the affected shoulder that had not responded to conservative treatment. The exclusion criteria included inability to speak or read English, previous shoulder surgery on the affected side, evidence of major joint trauma causing fracture, infection, underlying metabolic or inflammatory disease, avascular necrosis, frozen shoulder, major medical illness, and psychiatric illness that precluded informed consent. Patients with significant arthropathy and cuff tears extending into the subscapularis or teres minor were excluded from the study intra-operatively. All subjects provided an informed consent. Approval for use of human subjects was obtained from the Research Ethics Board of the Sunnybrook Health Sciences Centre and the University of Toronto.

### Operationalizing Sex and Gender

For the purpose of this study, "sex" referred only to biological and physiological differences in strength and passive range of motion. It was felt that the influence of exercise or training that might modify the biological qualities was minimal in the non-athlete sample included in our study. The term "gender" referred to non-biological aspects of being men or women such as "involvement in social activities or roles" and "expectations". These factors are influenced by social, cultural and economic factors. The following factors were examined as a product of an interaction between "sex and gender" that could not be studied in isolation: aging, extent of comorbidity, Body Mass Index (BMI), smoking, severity of bony and soft tissue pathology, incidence of work-related injuries, and pain perception that affects active range of motion. Body size and hormonal differences may increase susceptibility to injury in women. However, men and women have different life styles, risk taking behaviors, and pattern of health utilization [16,17]. Aging is not equivalent in men and women due to hormonal/biological and social/cultural differences, which together affect the overall life expectancy [18,19]. This discrepancy is particularly noticeable among countries with different levels of economic status, education and literacy [20,21]. Similarly, perception of pain severity is partly related to difference in neural and hormonal function [22,23] and partly related to social conditioning and cultural upbringing [24].

### Outcome Measures

In the present study, the primary outcome measure was a multidimensional disease-specific outcome measure, the Western Ontario Rotator Cuff (WORC) index [25] that was collected pre-operatively. The secondary self-report outcomes were collected for descriptive purposes and included the American Shoulder & Elbow Surgeons (ASES) assessment [26] and the Quick Disabilities of the Arm, Shoulder and Hand (*Quick*DASH) [27].

The WORC index consists of 21 items, each with a visual analogue scale type response option. This measure has five domains: 1) physical symptoms (6 questions); 2) sports and recreation (4 questions); 3) work (4 questions); 4) life style (4 questions); and, 5) emotions (3 questions). The highest or most symptomatic score is 2100 and the best or asymptomatic score is 0. In order to present this in a more clinically meaningful format, the score is reported as a percentage derived by subtracting the total from 2100, dividing by 2100 and multiplying by 100. The scores of the ASES and *Quick*DASH range from 0 to 100. While, 0 is the most symptomatic score for the ASES, it represents the least symptomatic score for *Quick*DASH. All disability measures (WORC, ASES, and *Quick*DASH) have been reported to be reliable and valid in patients with shoulder or rotator cuff pathologies [27-31]

The extent of participation limitation was measured by using one of the disability questions of the *Quick*DASH [27]. In this question, the interference of the upper extremity problems with participation in social activities is recorded in five categories on a 5-point Likert scale, "not at all", "slightly" "moderately", "quite a bit" and "extremely".

Patients' expectations for recovery were also documented subjectively. The expectation questionnaire included seven questions relating to pain relief, range of motion, activities of daily living, work, sports or leisure activities, interacting and providing care for others and overall expectation for recovery following surgery. Answers were quantified on a 5-point Likert scale. This questionnaire has shown discriminate validity in patients with rotator cuff pathology [32] and patients with osteoarthritis of the knee [33]. To determine homogeneity among the seven expectation questions, the Cronbach's alpha was calculated for the sample used in this study. The overall raw alpha (0.62) and inter-item correlations values were low (0.29 – 0.46) indicating that the expectation questions did not measure the same construct and hence each question was analyzed separately. All questionnaires were completed 2–3 weeks pre-operatively.

### Demographic, History-Related, Clinical, Radiological and Surgical Factors

Demographic data that were examined in relation with disability included age, Body Mass Index (BMI), hand dominance, affected side, side operated on, and co-morbidity. The BMI of less than 24.9 kg/m^2 ^was categorized as normal. Individuals with a BMI between 25 and 29.9 kg/m^2 ^were classified as overweight and the BMIs ≥ 30.0 indicated obesity [34]. Extent of co-morbidity in 13 systems was examined by the Cumulative Illness Rating Scale [35].

Variables related to history that were examined in relation to disability were medication use (yes/no), symptom duration, work status (having an active work-related claim), and smoking habits (yes/no). Clinical examination of the shoulder included strength, and active, passive, and painfree combined range of motion (flexion, abduction, external and internal rotation) as described by Constant and Murley [29,36]. The painfree range of motion represented the functional range of motion and ranged from 0 to 40, with 0 being the most restricted and 40 being the full score. Strength measurement in the scapular plane and 90 degrees of elevation was conducted by a simple unsecured tensiometer. The details of clinical assessment and scoring of the ROM and strength have been previously published [29]. The extent of bony pathology was examined radiologically. The information on existence of subacromial spurs, superior migration of humeral head, calcified tendinitis, osacromiale, and degenerative changes in the acromioclavicular (AC) and glenohumeral joints was taken from the radiologist's report. Existing pathological features in the report were recorded as 'yes' in the extraction data collection form, while normal findings were recorded as 'no'.

All patients underwent open or arthroscopic procedures based on the surgeon's preference. Patients with minor pathology in the rotator cuff tendons underwent arthroscopic or open decompression. Patients with full thickness tears of the rotator cuff underwent arthroscopic or open repair of the tendon(s). Some procedures overlapped (i.e. some patients underwent both repair and acromioplasty). Pathology in the AC joint, including osteolysis of the clavicle was also documented intra-operatively. Size of tear (largest dimension) was categorized as small < 1 cm, moderate (1–3 cm.), large (> 3–5 cm.), and massive (> 5 cm.). Strength, range of motion, level of pathology (existence of a full-thickness tear), concurrent osteoarthritis, and tear size were examined in relation with disability.

### Statistical Analysis

Sample size calculation was based on the primary outcome measure, the WORC. Based on pilot data (mean baseline WORC for females: 1441.8, SD: 384 and for males: 1340.5, SD: 425) and using a 2-sided test with an α value of 0.05 and power of 0.80, minimum of 140 patients (70 in each sex group) were required to detect a clinically important difference of 12% between men and women. Overall differences between men and women were examined by parametric and non-parametric statistics for continuous data and chi-squares and Fisher's Exact tests for categorical data as appropriate. Effect sizes were calculated for continuous data and interpreted using Cohen's classification [37].

In this study, for gender-sensitive analysis, guidelines proposed by Moerman and van Mens-Verhulst [38] were followed. Initially, descriptive statistics were calculated for all relevant variables for men and women separately. The univariable analyses examined the relationship between individual baseline variables and the primary outcome (WORC), which represented disability in the entire sample. To evaluate heterogeneity in men and women, these variables were further studied in each sex subgroup. The final analysis involved a multivariable analysis using ordinary least squares to assess the relationship between relevant baseline variables that were statistically significant at p < 0.1 in the univariable analysis. A multivariable analysis exposes separate components of biological, psychological or social origin that are integrated in the binominal variable of "sex". In such situations, the significance of the variable disappears when factors that represent "sex" and "gender" are entered into the equation.

Assumptions of multivariable analysis were examined. Multicollinearity among the independent variables was assessed and if the correlation (Pearson for continuous variables and Spearman's rho for ordinal variables) was greater than 0.75, then only one of the independent variables was selected [39]. Plausible interactions were examined among variables. Outliers related to categorical data (expectations) were collapsed with larger subcategories based on clinical judgment. Categories related to participation restriction were collapsed into three categories of low, moderate and high. The "low" category represented "not at all or slight interference", the moderate category represented "moderate interference" and "quite a bit or extremely" represented "high interference". Statistical analysis was performed using SAS^® ^version 9.1.3 (SAS^® ^Institute, Cary, NC). Statistical results are reported using 2-tailed p values with significance set at p < 0.05 or 0.01 for multiple comparisons.

## Results

One hundred and eighty five (91 females and 94 males) patients were recruited into the study. Fifteen patients were excluded intra-operatively due to having massive tears (3 females and 4 males) or arthropathy (3 females and 5 males). Data on 170 patients (mean age: 57, SD: 11, range: 32–87, 85 females, 85 males) were used for analysis.

### Overall Differences between Men and Women

Table 1 demonstrates the relevant demographic data and clinical examination results. Women were slightly older (p = 0.013). Men appeared to be more overweight where women appeared to be more in the normal or obese categories (p = 0.011). Men had a higher frequency of trauma to the shoulder (p = 0.03). The extent of comorbidity, smoking habit, symptom duration, medication use, and type of symptoms expressed by patients were not statistically significantly different between men and women. Similarly, the radiological findings reported by the radiologist and tear size documented intra-operatively were not statistically significantly different between men and women (Table 1). Intra-operative assessment of the AC joint showed slight differences with men having a higher frequency and severity of pathology. Overall, 94 patients had rotator cuff repairs without any significant differences between sexes (44 women and 50 men, p = 0.35). One female and one male had a repair of a deep partial thickness tear of the supraspinatus tendon. There was a difference in frequency of lateral resection of the clavicle, which was related to lower severity of AC joint arthritis in women and a higher incidence of osteolysis of the end of clavicle in men. Acromioplasty was performed more frequently in men. Biological differences were statistically significant between sexes with women having lower levels of strength (p < 0.0001). Active flexion (p = 0.001) and abduction (p = 0.002) and combined painfree range of motion (p = 0.009) were reduced in women while passive movements were similar in both sexes (p > 0.05). In terms of participation in social activities, a larger proportion of women reported "high interference" in their social functioning as compared with men who reported mostly low or moderate limitations (p = 0.002).

**Table 1:** Demographic and baseline characteristics (N = 170)

**Variables**	**Women(%)**	**Men(%)**	**Statistics P values**
**Age (Mean, SD)**	59.00 (11.07)	54.85(10.47)	**t value: 2.51** **p = 0.013**
Range	36–87	32–78	ES:0.39 (0.08–0.69)

BMI			
• Normal < 25	22 (26%)	11 (13%)	**χ^2^: 9.027**
• Overweight (25.0–29.99)	26 (30%)	44 (52%)	**P = 0.011**
• Obese (≥ 30.0)	37 (44%)	30 (35%)	

Comorbidity (Mean, SD)	3.34 (2.82)	2.61 (2.54)	t value: 1.78 p = 0.0764

Smoking			
• Yes	9 (11%)	16 (19%)	χ^2^: 2.29
• No	76(89%)	69 (81%)	p = 0.1295

Hand Dominance			
• Right	78 (92%)	77 (91%)	Exact Fisher's: 0.10
• Left	7 (8%)	7 (8%)	p = 1.00
• Bilateral	None	1 (1%)	

Affected Side			
• Right	48 (57%)	42 (49%)	χ^2^: 4.90
• Left	14 (16%)	26 (31%)	p = 0.086
• Bilateral	23 (27%)	17 (20%)	

Side operated on			
• Right	61 (72%)	50 (59%)	χ^2^: 3.14
• Left	24 (41%)	35 (41%)	p = 0.076

Symptom duration in months (Mean, SD)	45.06(71)	47.98 (60)	t value: -0.29 p = 0.77

Symptoms characteristics			
• Pain on movement	66 (78%)	67(79%)	χ^2^: 0.0345, p = 0.85
• Night pain	59 (69%)	51(60%)	χ^2^: 0.0, p = 1.00
• Weakness	57 (67%)	56 (66%)	χ^2^: 0.0, p = 1.00
• Catching/Clicking/Grinding	40 (47%)	40 (47%)	χ^2^: 0.0, p = 1.00

Extent of bony pathology			
• **AC joint arthritis**	**55(65%)**	**67(74%)**	**χ^2^: 4.18, p = 0.04**
• GH Arthritis	22(26%)	23(27%)	χ^2^: 0.05, p = 0.81
• Superior migration of humeral head			
• Subacromial Spurs	22(26%)	23(27%)	χ^2^: 0.03, p = 0.86
• Calcified Tendinitis	77(91%)	78(92%)	χ^2^: 0.7, p = 0.87
• Osteolysis of end of clavicle	5(6%)	6(7%)	χ^2^: 0.09, p = 0.76
• Osacromiale	0(0%)	3(4%)	Fisher's test: 0.12, p = 0.24
	1(1%)	1(1%)	Fisher's test: 0.50, p = 1.00

Mechanism of injury			
• Insidious	29 (34%)	23(27%)	χ^2^: 0.99, p = 0.31
• Repetitive activities	14(17%)	13 (15%)	χ^2^: 0.44, p = 0.83
• Fall	15 (18%)	10 (12%)	χ^2^: 1.172, p = 0.28
• **Traumatic**	**10 (12%)**	**21 (25%)**	**χ^2^: 4.77, p = 0.03**

Work Status (active work-related claim related to shoulder): 36/170	20(24%)	16(19%)	χ^2^: 0.56, p = 0.45

Type of surgery			
• Rotator Cuff repair	44 (52%)	50 (59%)	χ^2^: 0.85, p = 0.35
• **Resection of lateral Clavicle**	**49(58%)**	**63 (74%)**	**χ^2^: 5.13, p = 0.02**
• **Acromioplasty**	**77 (91%)**	**84 (99%)**	**Exact Fisher's: 0.015** **p = 0.03**

Size of full-thickness tear			
• Small	2 (2%)	2 (2%)	Exact Fisher's: 0.65
• Moderate	31 (33%)	36 (38%)	p = 0.86
• Large	12 (13%)	11 (12%)	

Medication taken			
• Yes	32 (39%)	41(47%)	χ^2^: 1.94, p = 0.16
• No	53 (61%)	44(52%)	

**Participation limitation**			
• Low	31 (36%)	45 (53%)	
• Moderate	18 (21%)	25 (29%)	**χ^2^: 12.36, p = 0.002**
• High	36(42%)	15(18%)	

**Strength (Mean, SD)** **(Elevation in scapular plane, lb)**			
• **Affected side**	**2.89(2.89)**	**7.01 (4.76)**	**-6.03 p < 0.0001**
• **Opposite side**	**7.81(3.28)**	**13.31(5.49)**	**-7.06 p < 0.0001**

**Range of motion (Mean, SD)**			
**Active flexion (0/180)**	**119 (42)**	**139 (39)**	**-3.23 0.001**
Passive flexion (0/180)	149 (29)	156 (25)	-1.43 0.1528
**Active abduction (0/180)**	**106 (47)**	**128 (43)**	**-2.91 0.002**
Passive abduction (0/180)	138 (37)	147 (30)	-1.36 0.17
Active external rotation (0/90)	46(19)	54(35)	-1.11 0.270
Passive external rotation (0/90)	56(19)	71(25)	0.977 0.329

**Combined painfree range of motion (0/40)**			
(Mean, SD)			
Flexion: 10			
Abduction: 10	**19.07 (9.61)**	**23.08 (9.92)**	**-2.65 0.009**
External Rotation: 10			
Internal Rotation: 10			

AC: Acromioclavicular

BMI: Body Mass Index

GH: Glenohumeral

SD: Standard Deviation

χ^2^:Chi-Square

No difference was detected between genders with respect to their expectations for improved pain, ADL, sports/recreational activities, and achieving full recovery (Table 2). However, women were different in terms of their expectations in interacting and providing care for others, improved range of motion, and return to work. Women had higher expectations for improved ability to provide care to others (p = 0.003). A larger proportion of men however, reported no difficulty with this task. In terms of expectations for improved range of motion, a larger proportion of men expected full recovery of range of motion (p = 0.023). More men were working with or without discomfort and more women were on disability (p = 0.006), on unemployment, or retired without a significant difference between their expectations for return to full time or part time work.

**Table 2:** Gender-related differences with respect to expectations for recovery

**Variables**	**Pr <= P **
Do you expect your surgery to help with **pain relief**?	0.802
Do you expect your surgery to increase your painfree **range of motion**?	**0.023**
Do you expect your surgery to improve your **ability to carry out the normal activities of daily living**?	0.370
Do you expect your surgery to improve your **ability to care for others**?	**0.003**
Do you expect to **return to work **following your surgery?	**0.006 **
Do you expect that following your surgery you will be able to participate in the **leisure, sports, or recreational activities **you did before your problem started?	0.086
Do you expect that following your surgery the area operated upon will be **back to the way it was **before you began having problems there?	0.863

Women reported higher levels of disability as defined by the primary (WORC) and secondary subjective outcome measures (ASES, and *Quick*DASH) (Table 3). The effect sizes varied from small to moderate (0.37 to 0.61). The Sub-domains of the WORC, "life style" and "work", which reflect sleeping, daily activities and routinely performed tasks (i.e. overhead movements, lifting, styling hair, dressing) demonstrated higher disability scores for women.

**Table 3:** Differences in men and women in primary outcome, the WORC and its sub-domains and secondary outcomes, the ASES and *Quick*DASH

**Variables (Min/Max)**	**Women** **Mean ** **(SD)**	**MEN** **Mean** **(SD)**	T/Z values **	**P values**	**Effect Size (CI)**
**WORC Total Raw score (0/2100)**	1368.58	1234.12	**2.42**	**0.017**	0.37
*Higher numbers mean more disability*	(371.74)	(353.03)			(0.07–0.67)
**WORC Percentage (0/100)**	34.84%	41.22%	**-2.42**	**0.017**	0.37
*Higher numbers mean less disability*	(17.70)	(16.80)			(0.07–0.67)

**Domains of the WORC**					
Symptoms (0/600)	351.88	326.58	1.36	0.175	
	(130.22)	(111.05)			
Life style* (0/400)	271.10	223.35	**3.99**	**< 0.0001**	0.58
	(88.67)	(84)			(0.27–0.88)
Work* (0/400)	284.55	249.60	**3.25**	**0.001**	0.47
	(71.12)	(80)			(0.16–0.77)
Sports/recreational activities* (0/400)	290.38	282.45	1.077	0.282	
	(70.92)	(67.43)			
Emotions (0/300)	170.59	153.96	1.42	0.157	
*Higher numbers mean more disability*	(73.23)	(79.12)			

**ASES (0/100)**	**42.92 **	**51.12 **	**-2.77**	**0.0062**	**0.42**
*Higher numbers mean less disability*	**(21.28)**	**(16.99)**			(0.12–0.73)

***Quick *DASH (0/100)**	**55.82 **	**44.87**	**3.98**	**0.0001**	**0.61**
*Higher numbers mean more disability*	**(18.77)**	**(17.10)**			(0.30–0.92)

T Test (T values): used for normally distributed data

*Wilcoxon-Mann-Whitney test (Z values): used for skewed data

CI: Confidence interval

**To adjust for multiple comparison of 5 domains, the p values were adjusted by a' = a/k, where a = 0.05, k = the number of multiple comparison variables: 0.05/5 = 0.01

Effect size values are reported for significant differences: Small (0.20–0.49), Moderate: (0.50–0.79), Large: > 0.80 (Cohen, 1988).

### Univariable Analysis: Relationship Between disability and independent variables

Table 4 shows the results of the univariable analysis. Among independent variables (age, BMI, comorbidity, smoking, hand dominance, affected side; side operated on, symptom duration, mechanism of injury, strength, range of motion, existence of a full-thickness tear, concurrent osteoarthritis, tear size, medication use, work status, participation restriction, and expectations) that were examined in relation to disability as defined by the WORC, painfree combined range of motion, strength, and participation limitation had a positive relationship with disability (more limitation correlated with more disability). Smoking, use of medication, having bilateral shoulder problems, and having a work-related injury (on compensation benefits) were associated with higher disability scores. In terms of expectations, patients who did not have any difficulty with interacting and providing care for others, carrying out their normal ADL, or had no loss of painfree range of motion were significantly less disabled than those who expected improvement. Expectations with respect to paid work showed the highest contrast between working and non-working patients. The least disabled people were those who were working with or without discomfort, while the highest reported disability was observed in those who expected a return to modified/part time work.

**Table 4:** Univariable Analyses: The association between level of pre-operative disability and each independent variable

**Independent variables**	**DF**	**R-Square **	**F**	**P value**
**Binominal factor of man/woman**	**1**	**0.033**	**5.85**	**0.017**

Age	1	0.014	2.38	0.125

BMI	2	0.015	0.26	0.288

Comorbidity	1	0.0005	0.09	0.769

Mechanism of injury	3	0.0158	0.75	0.524

Hand dominance	1	0.0033	0.31	0.735

**Affected side**	**2**	**0.0327**	**3.06**	**0.049**

Side operated on	1	0.0001	0.02	0.894

Symptoms duration	1	0.0069	1.26	0.264

**Combined painfree ROM**	**1**	**0.305**	**72.69**	**< 0.0001**

**Strength (operated side)**	**1**	**0.120**	**22.19**	**< 0.0001**

**Smoking**	**1**	**0.025**	**4.27**	**0.040**

Concurrent Osteoarthritis (Glenohumeral arthritis/humeral head migration)	1	0.001	0.24	0.627

Repair vs. no repair	1	0.006	0.97	0.325

Size of full-thickness tear (Small, moderate, large)	2	0.003	0.15	0.864

Use of medication	**1**	**0.030**	**5.16**	**0.024**

**Work status (active work-related injury)**	**1**	**0.071**	**12.91**	**0.0004**

**Participation limitation**	**2**	**0.396**	**55.19**	**< 0.0001**

Expectations with respect to improved pain	1	0.004	0.07	0.7895

Expectations with respect to improved ROM	2	0.024	2.05	0.1326

**Expectations with respect to improved ADL**	**2**	**0.093**	**8.46**	**0.0003**

**Expectations with respect to improved interaction and providing care**	**2**	**0.119**	**11.03**	**< 0.0001**

**Expectations with respect to return to work**	**3**	**0.142**	**6.74**	**< 0.0001**

Expectations with respect to return to sports	2	0.021	1.77	0.173

Expectations with respect to achieving full recovery	2	0.014	1.18	0.311

### Subgroup-Analysis

Subgroup analysis of the randomized controlled studies would produce conclusive results when it is based on apriori hypothesis with sufficient number of subjects in each group and existence of interaction between treatment effect and risk factors [40-42]. In observational studies this type of analysis is usually hypothesis generating and helps to identify the difference in strength and direction of the relationship between outcome and independent variables in each group. The subgroup analyses of the primary outcome based on men and women showed consistency between the overall effect and the differential subgroup effect with a similar pattern of relationship between the WORC and independent factors in majority of the cases except for age, having a repair, medication use, and expectations for improved ADL and ROM (Table 5). The relationship between disability and age, taking medication, and having a full-thickness tear that required a repair showed an interesting dissimilarity between men and women. Younger men reported more disability than older men while age did not have a significant association with disability in women. Similarly, men with a full-thickness tear who underwent a repair were significantly more disabled than men who did not have a repair (a mean difference of 209 in WORC scores). Women however, were not statistically significantly different (a mean difference of only 73 WORC scores between repair and no repair groups). Taking medication had a reversed pattern indicating that women who were taking medications were more disabled. Women in the "not applicable category" who had no problem with their ADL activities were significantly less disabled than those who expected improvement, while men's disability did not have a strong relationship with their expectations, being fairly close among those with no complaints and those who expected improvement. The same pattern was observed for expectations for improved ROM. The differences in other factors (affected side, work status, participation limitation, and expectations for improved care/interaction and return to work) with respect to disability were in the same direction but of slightly different magnitudes.

**Table 5:** Subgroup analyses

**Independent Variables**	**R-Square** Women/Men	**F value** Women/Men	**P value** Women/Men
Age	0.008/**0.054**	0.70/**4.80**	0.407/**0.031**

BMI	0.007/0.026	0.28/1.11	0.758/0.335

Comorbidity	0.001/0.0002	0.11/0.20	0.740/0.652

**Combined Painfree ROM**	**0.326/0.249**	**38.81/27.66**	**< 0.0001/< 0.0001**

**Strength (operated side)**	**0.083/0.099**	**7.56/9.18**	**0.007/0.003**

Smoking	0.030/0.038	2.55/3.30	0.114/0.073

Mechanism of injury	0.010/0.064	0.22/1.44	0.884/0.238

Hand dominance	0.007/0.007	0.59/0.29	0.445/0.746

Affected side	0.043/0.024	1.88/1.03	0.159/0.363

Side operated on	0.011/0.001	0.94/0.10	0.336/0.753

Symptoms duration	0.007/0.001	0.62/0.10	0.424/0.754

Concurrent osteoarthritis	0.030/0.010	2.77/0.85	0.099/0.359

Level of pathology	0.006/**0.086**	0.97/**7.82**	0.325/**0.006**

Size of full-thickness tear	0.024/0.005	0.53/0.12	0.594/0.886

Use of medication	**0.048/**0.008	**4.22/**0.71	**0.043/**0.402

**Work Status (work-related injury)**	**0.059/0.080**	**5.18/7.25**	**0.025/0.009**

**Participation limitation**	**0.338/0.430**	**21.02/31.07**	**< 0.0001/< 0.0001**

Expectations with respect to improved pain	0.001/0.060	0.12/2.56	0.726/0.083

Expectations with respect to improved ROM	**0.102/**0.001	**4.57/**0.11	**0.013/**0.739

Expectations with respect to improved ADL	**0.227/**0.025	**12.05/**0.99	**< 0.0001/**0.376

**Expectations with respect to improved interaction and providing care**	**0.125/0.094**	**5.59/4.05**	**0.005/0.021**

**Expectations with respect to return to work**	**0.130/0.163**	**3.99/5.07**	**0.011/0.003**

Expectations with respect to return to sports	0.012/0.036	0.48/1.45	0.618/0.240

Expectations with respect to achieving full recovery	0.008/0.042	0.36/1.79	0.697/0.174

### Multivariable Analysis: Relationship between Disability and all Significant Independent Variables

The multivariable analysis showed that the impact of the binominal factor of sex that included both sex and gender qualities, disappeared after incorporating factors that represented sex and factors that signified gender. Three factors remained significant in multivariable regression of the WORC; painfree range of motion, participation limitation and expectations for improved ability of carrying out the normal activities of daily living (Table 6).

**Table 6:** Multivariable Analysis

**Independent variables**	**DF**	**β Estimates**	**F value**	**P value**
Binominal factor of sex	1	Female: 1.86	0.67	0.4160
		Male: 0.00		

**Participation limitation**	2	Low: 17.84	**28.91**	**< 0.0001**
		Moderate: 7.93		
		High: 0.00		

**Combined painfree ROM**	1	0.65	**25.82**	**< 0.0001**

Strength	1	0.07	0.07	0.7945

Smoking	1	Yes: 3.09	0.00	0.9972
		No: 0.00		

Work Status (active work-related injury)	1	Yes: -2.13	0.76	0.3846
		No: 0.00		

Affected side	2	Bilateral:-3.80	2.45	0.0900
		Left: 1.76		
		Right: 0.00		

Medication use	1	Yes: -1.10	0.33	0.5673
		No: 0.00		

Expectations with respect to improved interaction and providing care	2	No difficulty: 2.63	1.30	0.2746
		Moderate expectations: -1.59		
		High expectations: 0.00		

**Expectations for improved ADL **	2	No difficulty: 16.93	**5.80**	**0.0038**
		Moderate expectations: 2.12		
		High expectations: 0.00		

Expectations with respect to return to work	3	Not applicable: 3.69	1.00	0.3929
		Working: 2.64		
		Light: -1.08		
		Full: 0.00		

**Full Model**	**17**	**R-Square: 0.63**	**14.26**	**< 0.0001**

## Discussion

Identifying sex and gender related determinants of musculoskeletal health and their complex interactions is becoming a priority for all researchers who hope to have a more accurate measure of disability. In the present study, with gender-sensitive analyses, the binominal variable of sex was decomposed into separate components of biological and psychological/social origin. Factors that were associated with disability represented either gender or an interaction between sex and gender.

Our results add to the only previous study [13] that has examined gender differences in patients with rotator cuff pathology as the main objective. In the previous study, the WORC total score was not significantly different but the domain related to emotions was different between men and women [13]. The difference between the present study and the previous one is a difference in the analytical approach. In the previous study [13], disability scores were divided into two categories based on the median, which could affect the sensitivity of the measurement. With a more rigorous approach to data collection and analysis, it was found that disability was affected by a large number of biological, social and psychological factors that distinguish women from men. In terms of extent of soft tissue pathology, the previous study reported a slightly higher prevalence (p = 0.036) of smaller tears in female patients less than 55 years of age, but not in older women. In our study, the number of patients with small tears was not sufficient to examine age differences between men and women. Similarity of the findings of the present study with the previous study is related to the impact of aging on disability (aging reduces the level of reported disability), which upon closer examination appears to be affecting men and women differently. Only one other study [14] examined differences between men and women and was limited due to a small sample and sub-optimal analysis. The authors examined 72 patients with full-thickness tears (44 men and 28 women). They used separate non-parametric correlation analyses and examined the relationship between age, tear size, and scores of three subjective shoulder outcomes [Constant-Murley, Simple Shoulder Test (SST), and University of California Los-Angeles (UCLA)] in patients suffering from rotator cuff pathology [14]. They reported a low negative correlation between tear size and scores of the SST and Constant-Murley outcomes in both sexes (women: -0.35 and -0.35; men: -0.43 and -0.49 respectively) and a negative correlation between subjective scores and age in women but not in men. Our result in terms of impact of age on disability was in the opposite direction with younger men being the most disabled group. Apart from the low and insignificant correlation coefficients [14], subgroup analysis is usually not conclusive in observational studies due to lack of control group. Conducting exploratory subgroup analysis on small samples in the absence of overall treatment effect is prone to error in interpretation.

In terms of physical impairment, Bassey and colleagues [15] reported lower level of abduction, which is in agreement with our results. Women on average had less strength than men. Despite similar levels of passive range of motion, women were more hesitant to move beyond the painful range which might have been due to fear of pain rather than a purely biological difference, yet another interaction between sex and gender.

In our study, participation in social activities had the strongest independent association with disability. Participation limitation in women has not been explored extensively in the literature. However, consistent with our results, Müllersdorf and Söderback [43] who examined this aspect of disability in Swedish individuals with disabilities reported that women were more affected in their daily and work activities.

We found that expectations for improved ADL were statistically significantly related to disability as defined by the WORC. There is limited information on expectations in patients with shoulder complaints [44,45]. In one study that involved patients with rotator cuff pathology, the authors [44] adjusted for sex and therefore it is not clear if men and women had a different level of expectation. Adjusting for sex without examining the impact of such adjustments on the analysis could lead to faulty conclusions. By adjusting or controlling for sex, one presumes that women have the condition of interest more often than men because of hormonal or other biological factors. Beginning with this assumption, makes it less likely that a relation between musculoskeletal conditions and women's roles in family/society will be detected. The results of other study [45] indicate some gender differences in expectations with women having higher levels of expectations for improved activities of daily living. This is consistent with our analysis of group and subgroup differences that women indeed are more concerned about their activities of daily living and providing care for others. Obviously, women have a primary role in family and their responsibilities within the household (e.g. childcare, household chores) would explain these gender differences. Moreover, social traditions, customs, and obligations create different expectations and constraints for female patients.

The purpose of gender-sensitive studies is to improve the overall diagnostic process and interpretation of the statistical analysis which has implications in terms of providing equal opportunities, services and programs leading to better treatment for both men and women. The specific implication of the finding related to pain-free range of motion is to facilitate the rehabilitation needs of women with rotator cuff pathology particularly if they are engaged in jobs that involve repetitive overhead activities (by providing sex-based rehabilitation that accounts for women's unique structural and biological differences). It may be beneficial to accommodate ergonomic assessments to identify and reduce risk factors which may pose differential biomechanical stresses to the female workers.

In terms of gender-specific differences (expectations and participation limitations), women's unique care-giving roles in family and society make them more susceptible to disability as they need to fulfill more responsibilities and expectations compared to their male counterparts. To reduce disability secondary to rotator cuff disease, these socially-oriented factors need to be considered. This study was not designed to measure disparity in access to care. However, improving women's access and affordability (prioritization of females by decreasing the waiting period to see a physical therapist, occupational therapist, or orthopaedic surgeon and increasing the frequency or number of treatments, etc.), may be effective in reducing disability in female patients with rotator cuff pathology. Most importantly, by giving better access to external social resources to those who provide care to small children or older individuals at home, women's recovery after rotator cuff surgery will be accelerated.

### Limitations

In the present study despite a large of number of factors examined, certain important gender related differences such as marital status, level of income, having dependent children, and extent of family and social support were not explored. Future studies should acknowledge the importance of the above factors and other social, cultural, and economic determinants of health. More sensitive measures of participation are needed to capture the important aspects of involvement in life situations and factors that influence that. Longitudinal studies will add to our understanding of how these factors affect the overall recovery from surgery in patients suffering from rotator cuff related pathologies.

## Conclusion

The findings of the present study indicate that male and female candidates for rotator cuff surgery have similar levels of bony (with the exception of the acromioclavicular joint) and soft tissue pathologies, comorbidity, and symptom characteristics. However, women report suffering from higher levels of disability due to their unique biological and non-biological differences in pain-limited range of motion, participation in social and family activities and expectations for recovery. Considering the significant impact of non-biological factors on disability, identifying gender related differences may help clinicians to direct their focus on what matters most to the patients suffering from rotator cuff pathology.

## Competing interests

The authors declare that they have no competing interests.

## Authors' contributions

This study was conducted in partial fulfillment of the requirements for the degree of Doctor of Philosophy for HR. HR conceived the idea, wrote the protocol, performed the clinical examination, supervised data collection and entry, conducted the analysis, and drafted the manuscript. AMD and SBJ co-supervised the protocol development, statistical analysis, and edited the manuscript. RH and RRR performed the surgical procedures and provided input on study design, protocol development and the manuscript. RRR was the faculty supervisor of the PhD thesis. All authors have read and approved the final manuscript.

## Pre-publication history

The pre-publication history for this paper can be accessed here:


